# From Stem Cells to Bone-Forming Cells

**DOI:** 10.3390/ijms22083989

**Published:** 2021-04-13

**Authors:** Samantha Donsante, Biagio Palmisano, Marta Serafini, Pamela G. Robey, Alessandro Corsi, Mara Riminucci

**Affiliations:** 1 Department of Molecular Medicine, Sapienza University of Rome, Viale Regina 324, 00161 Rome, Italy; samantha.donsante@gmail.com (S.D.); biagio.palmisano@uniroma1.it (B.P.); alessandro.corsi@uniroma1.it (A.C.); 2 Centro Ricerca M. Tettamanti, Clinica Pediatrica, Università di Milano-Bicocca, Ospedale San Gerardo, 20900 Monza, Italy; serafinim72@gmail.com; 3 Skeletal Biology Section, National Institute of Dental and Craniofacial Research, National Institutes of Health, Department of Health and Human Services, Bethesda, MD 20892, USA; probey@dir.nidcr.nih.gov

**Keywords:** osteoblasts, skeletal stem cells, bone, bone marrow stromal cells, skeletal biology

## Abstract

Bone formation starts near the end of the embryonic stage of development and continues throughout life during bone modeling and growth, remodeling, and when needed, regeneration. Bone-forming cells, traditionally termed osteoblasts, produce, assemble, and control the mineralization of the type I collagen-enriched bone matrix while participating in the regulation of other cell processes, such as osteoclastogenesis, and metabolic activities, such as phosphate homeostasis. Osteoblasts are generated by different cohorts of skeletal stem cells that arise from different embryonic specifications, which operate in the pre-natal and/or adult skeleton under the control of multiple regulators. In this review, we briefly define the cellular identity and function of osteoblasts and discuss the main populations of osteoprogenitor cells identified to date. We also provide examples of long-known and recently recognized regulatory pathways and mechanisms involved in the specification of the osteogenic lineage, as assessed by studies on mice models and human genetic skeletal diseases.

## 1. Introduction

Many advances have been made over the last few years in the field of bone biology. They have not overtly changed our concept of bone-forming cells, which has remained firmly rooted in classic topographic, morphological, and functional criteria, but have led to the emergence of new and exciting paradigms regarding their origin and differentiation. In this review, we place special emphasis on the concepts and findings that have a significant influence on our approach to bone pathology. By referring to mouse models and human genetic skeletal disorders, we also highlight the impact that the evolving comprehension of the origin and specification of osteoblasts has on our understanding of bone diseases. Emerging concepts in skeletal stem cell biology and osteoblastogenesis are presented. We first summarize the steps that occur during bone development and continue with a discussion on the canonical bone marrow skeletal stem cell system and other cell types that have been recently identified as sources of bone-forming cells. Then, we describe some of the best-known molecular factors and mechanisms that regulate osteoblastogenesis during bone development and homeostasis, with a particular focus on the role of the stimulatory G protein (Gsα)-cyclic 3′,5′-adenosine monophosphate (cAMP) signaling pathway. Finally, we discuss new insights recently reported into the role played by osteoclasts as modulators of osteoblast differentiation.

## 2. Defining Bone-Forming Cells

Bone-forming cells, termed osteoblasts, are cells that produce the type I collagen-enriched extracellular matrix found in the skeleton. Their activity is critical to skeletal morphogenesis and to the processes that occur in individual bone segments in order to achieve proper size and shape (growth and modeling), to maintain adequate mass and structure (remodeling), and to restore normal tissue composition and architecture after injury (regeneration) [1] The main function of osteoblasts is to produce and organize the extracellular bone matrix through the expression of a wide panel of genes that encode structural, enzymatic and regulatory proteins (collagen type I (COL1A1, COL1A2), alkaline phosphatase (ALP/TNAP), bone sialoprotein (BSP/IBSP), osteopontin (OPN/SPP1), osteonectin (ON/SPARC), osteocalcin (OCN/BGLAP) and others), which are commonly used as osteogenic markers [2]. Their secretory activity is controlled by a wide range of signaling pathways induced by growth factors, hormones, prostaglandins, cytokines, and vitamins [3,4]. However, osteoblasts themselves secrete a wide spectrum of molecules that act as autocrine, paracrine, and hormonal factors and are involved in the regulation of hematopoiesis, osteoclastogenesis, mineral homeostasis, and energy metabolism [4,5]. Thus, besides forming bone, osteoblasts regulate their own activity, the activity of their neighbors, establish a connection between the skeleton and other organs, and take part in a wide range of body functions.

**Figure 1:**
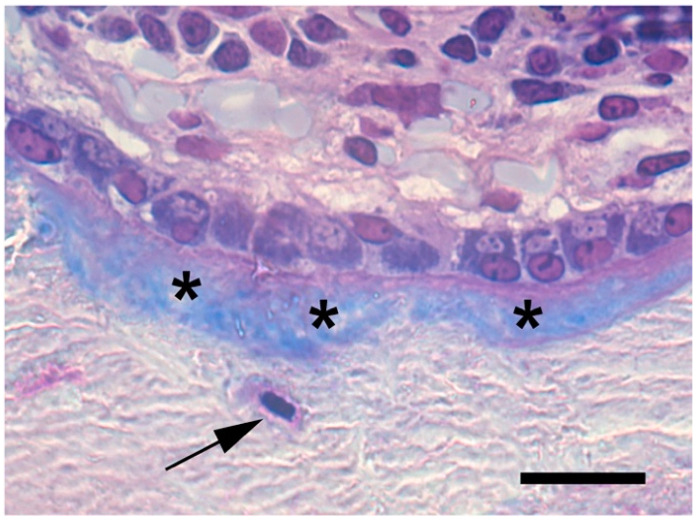
Osteoblasts rimming a bone trabecula with unmineralized (asterisk) and mineralized matrix with embedded osteocytes (arrow). Formalin-fixed, non-decalcified plastic-embedded tissue, May-Grünwald-Giemsa. Scale bar: 25 µm.

Osteoblasts are found on the surface of the newly formed bone, where they are easily identified as cuboidal cells (Figure 1) during skeletal formation, growth, and repair. At the end of their life span of approximately 3 months [6], osteoblasts may undergo apoptosis or complete their life cycle by converting into either of two differentiated cell phenotypes: osteocytes or bone lining cells. Osteoblasts being encased by an extracellular matrix (osteoid) that will become mineralized develop long dendritic-like cytoplasmic projections and become osteocytes, which represent the end stage of osteogenic differentiation, and may live as long as 50 years [6]. In contrast, those that remain on the bone surface assume a flat morphology, thus converting into bone lining cells [7], which in many species (e.g., birds and mice) may revert into osteoblasts during normal bone remodeling and in response to anabolic stimuli [8,9]. Due to their multiple functions and limited survival, osteoblasts must be continuously replenished by progenitor cells to maintain skeletal activities throughout life [10]. Studies in humans and mice have identified different lineages of osteoblast precursors during embryonic and fetal development as well as after birth. In addition, experimental evidence suggests that osteoblasts may also derive from hypertrophic chondrocytes. The reason for the existence of multiple sources of osteoblasts is still unclear. It does not seem to depend on space and/or time-specific requirements of bone formation as fully differentiated osteoblasts at different anatomical sites and in different phases of life share basic biological features and activities. Rather, it seems to be related to the complex developmental pattern [11] microenvironment, and functional adaptations that the skeleton undergoes before and after birth [12] that require the cooperation of different cohorts of progenitor cells with specific properties and tasks.

## 3. The Origin of Bone-Forming Cells in Developing Bones

**Figure 2:**
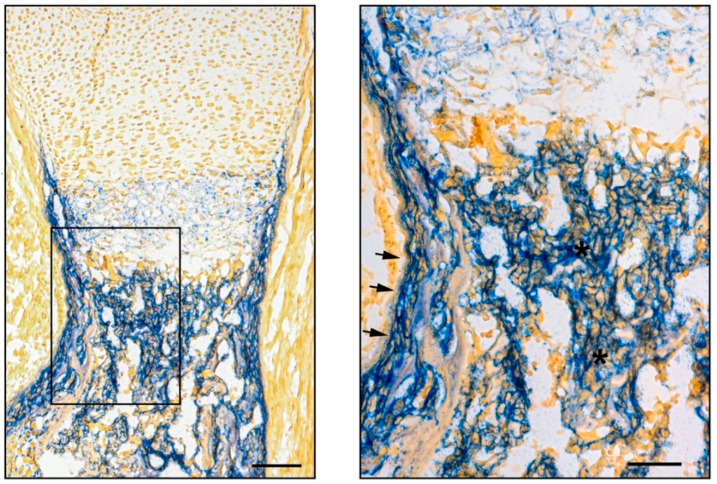
ALP histochemistry showing osteogenic cells in the periosteum (arrows) and within the marrow cavity (asterisks) in the developing femur of an E18 mouse embryo. Right panel represents the boxed region in the left panel. Formalin fixed tissue embedded in optimal cutting temperature (OCT) compound after decalcification. Scale bars: 100 µm and 50 µm in the left and right panel, respectively.

The mature skeleton is comprised of multiple tissues including cartilage, bone, marrow stroma, and marrow fat that appear during skeletal ontogeny in an asynchronous manner, with cartilage and bone being the first (by the end of the eight weeks after conception in humans) and marrow stroma and fat the last (in peri- and post-natal life). In the developing embryo, skeletal progenitors originate from two different germ layers: neuroectoderm, generating facial bones, and mesoderm, giving rise to the cranial vault and axial bones (paraxial mesoderm), and appendicular bones (lateral plate somatic mesoderm) [11]. In both layers, primitive mesenchyme condenses at sites of prospective skeletal development and generates the earliest matrix-forming cells that differentiate into either osteoblasts or chondrocytes [13]. The generation of osteoblasts that directly produce bone is known as intramembranous ossification and occurs during the morphogenesis of cranial bones surrounding the brain and brainstem, facial bones, the lateral part of the clavicle, and the periosteal collar of long bones. Studies on the expression of osteogenic markers such as ALP, OPN, and BSP in the cranial vault of mouse embryos suggest that in the first step of membranous ossification, progenitor cells expand outward from the cell condensation to define the primary region of osteogenesis and the early shape of bone [14,15]. Differentiation starts in the inner part where mature osteoblasts produce a collagenous network that rapidly mineralizes, while progenitor cells continue to proliferate at the border to ensure the expansion of the segment. This appositional pattern of growth that characterizes membranous ossification is designed for flat bone formation but not for tubular bones, for which a rapid gain in length is required during intra-uterine life and after birth until reaching skeletal maturity. The majority of the skeleton is formed by an alternative endochondral ossification process. In this case, the earliest matrix-forming cells are chondrocytes [13], which form a provisional cartilaginous mold that expands by appositional and interstitial growth, becomes hypertrophic, undergoes mineralization, and is eventually replaced by bone. In this developmental pattern, osteoprogenitor cells are induced to form in the perichondrium, which is the outermost layer of the cartilaginous anlagen that is thereby converted into the periosteum. These newly formed osteoprogenitors invade the hypertrophic cartilage along with blood vessels, forming the primary ossification center of the cartilage [16]. Thus, in long bone formation, osteoblasts are generated at the perichondral border, to produce intramembranous bone in the primitive bony collar, and within the anlagen, to replace the mineralized hypertrophic cartilage with trabecular bone (Figure 2). In both the intramembranous and endochondral processes, hypertrophic chondrocytes play a major role in osteogenesis by inducing the appearance of osteoprogenitor cells in the neighboring perichondrium [17], and by converting into bone-forming cells in the primary spongiosa [18]. In addition to producing osteoblasts, progenitor cells relocated from the perichondrium to within the developing bone marrow cavity provide a permanent cell coverage around blood vessels (termed pericytes, mural cells, or adventitial cells), and establish the marrow stroma, which is a locally adapted osteogenic tissue that supports hematopoiesis. When the marrow space exceeds the hematopoietic need, stromal cells convert into adipocytes. Meanwhile, stromal cells and adipocytes also appear in the marrow cavity of bones formed by membranous ossification, wherein they are generated by mesenchyme-derived perivascular cells. Adipocytes rapidly become a prominent marrow cell type in some skeletal sites (apophyses of long bones and terminal phalanges of hands and feet in humans, tail vertebrae and short bones in mice), and they progressively increase with growth and aging in the remaining skeletal segments.

## 4. The Origin of Bone-Forming Cells in Fetal and Adult Bones

### 4.1. Bone Marrow Skeletal Stem Cells

At the end of bone development, a new osteoprogenitor cell system evolves in the marrow cavity throughout the skeleton. In post-natal life, this system replenishes osteoblasts and marrow adipocytes but does not typically form chondrocytes under physiological conditions. Thus, compared to more primitive osteo-chondrogenic cells, osteo-adipogenic marrow progenitors better meet the needs of the adult skeleton to maintain the bone mass while providing a dynamic and flexible microenvironment for hematopoiesis. Most of our current knowledge on the biology of marrow osteoprogenitor cells was provided by Alexander Friedenstein along with Maureen Owen and coworkers, whose in vivo experimental models, based on heterotopic transplantation, still represent a mainstay of research in osteogenesis. Friedenstein first demonstrated that osteogenic precursors in marrow may be expanded ex-vivo as adherent bone marrow stromal cells (BMSCs) and then transplanted in vivo with an appropriate scaffold to generate heterotopic bone/marrow organoids (ossicles) including donor-derived bone, marrow stroma, adipose, and host-derived hematopoiesis [19]. Subsequently, he and others showed that some individual BMSCs can grow in a density-independent manner to produce a colony, initiated by a Colony Forming Unit-Fibroblast (CFU-F), and that the clonal progeny of some CFU-Fs are multipotent and reproduce a complete ossicle [20,21]. Further studies revealed that multipotent CFU-Fs also differentiate into chondrocytes when anaerobic conditions are established either in vivo (closed transplantation systems) [22] or in vitro (micro-mass cultures and cell pellets) [23]. Based on Friedenstein’s work, and with the emergence of so-called regenerative medicine in parallel, CFU-Fs and their progeny have become the subject of intense investigation as a potential tool for bone regeneration. However, their precise identity in vivo has remained unaddressed, and their effective stemness in vivo and ex-vivo long unproved. In all biological systems, stem cells are defined by the ability to self-renew their own compartment while generating functional differentiated offspring. For all biological systems, these two defining stem cell properties should ideally be assessed in vivo by cell lineage tracing and/or appropriate functional assays in vitro. Multiple challenges posed by the structural complexity and the very low turn-over of bone tissue, as well as difficulties in identifying suitable markers, have long hampered these types of studies in the bone field. Nonetheless, significant progress has been made over the last few years through combined approaches based on anatomic, phenotypic, and functional criteria (human and mouse), or through genetic labeling (mouse) (Table 1). In addition to the STRO1 antigen (now known to be HSC70) [24] originally reported by Simmons [25], other useful surface markers as melanoma cell adhesion molecule (MCAM)/CD146 [26], nerve growth factor receptor (NGFR)/CD271 [27], and platelet derived growth factor alpha (PDGFRα) and CD51/Integrin α_V_ [28] have been identified in human CFU-Fs. CD146 is expressed by perivascular CFU-Fs and its immunolocalization in the heterotopic ossicle has allowed confirmation of the self-renewal of multipotent CFU-Fs in vivo, hence the term bone marrow skeletal stem cells (bmSSCs) [26]. CD271 is also expressed by perivascular CD146^+^ CFU-Fs and by a population of CD146^−/low^ CFU-Fs residing in the marrow close to the endosteum [27]. PDGFRα^+^ and CD51^+^ identify a subset of CD146^+^ CFU-Fs found in fetal and, at a lower frequency, post-natal bone [28]. In mice, multiple populations of stromal cells with specific antigenic profiles and anatomical positions (e.g., association with blood vessels or nerve fibers) have been identified as progenitors of osteoblasts and, in some cases, of adipocytes (Table 1).

Although the precise phenotypic identity of human and mouse bmSSCs is still under investigation and much remains to be learned about their biology, it must be noted that these recent studies have brought at least two new important concepts into the bone field. The first reads that bmSSCs functionally interact with endothelial and hematopoietic cells to establish and maintain a functional marrow cavity [26,29,30]. This has provided a new perspective for the study of the pathophysiology of the hematopoietic niche/microenvironment. The second is the notion that a multipotent skeletal lineage unfolds from bone to marrow, establishing a physical and functional continuity between the two compartments. This has profoundly changed our approach to the pathology of the post-natal skeleton. Abnormalities in bone formation occurring after birth have long been interpreted exclusively in terms of defective function of differentiated osteoblasts and/or defective turnover of the extracellular matrix that they produce by osteoclasts. Based on these criteria, many bone diseases have escaped any reasonable pathogenetic explanation for years. The identification of a multipotent bmSSC has led to the appreciation that osteoblasts are part of a lineage and that bone diseases may result from malfunctioning of differentiated osteoblasts or from inherent or secondary malfunction of the entire lineage. The latter approach has clarified, at least in part, by the mechanisms underlying some diseases in which changes in bone formation are accompanied by concomitant abnormalities in marrow adipocytes and/or marrow stroma. This is well exemplified by osteoporosis, in which the reduction in the bone mass associates with the expansion of marrow adipocytes [31], or by secondary hyperparathyroidism and Fibrous Dysplasia of bone (FD, OMIM 174800), in which bone abnormalities are accompanied by the accumulation of stromal cells at endosteal sites and a complete fibrous conversion of bone marrow with loss of adipocytes and hematopoiesis-supportive stroma [32,33]. A major point that still remains to be addressed is how precisely the bmSSC lineage unfolds in vivo and what differentiation steps are undertaken by the osteogenically committed progeny before reaching a mature osteoblast stage. It is well known that the ontogeny of bone-forming cells recognizes at least three main phases as first suggested by Owen and colleagues [34], who followed the fate of rat periosteal cells after a ^3^H-thymidine pulse-labeling in vivo. Owen’s studies clearly showed that in the periosteum, pre-osteoblasts in the cambial layer, matrix-forming osteoblasts, and osteocytes represent subsequent stages in the lifespan of osteogenic cells, which are easily recognizable based on topographic and morphologic criteria. Within the bone marrow, a complex hierarchical organization emanating from the multipotent bmSSC has been initially inferred from the heterogeneity of individual stromal cell colonies in terms of proliferation and differentiation activity [20,21,35,36]. More recent studies performed in mice through single cell analysis have identified cell clusters within the marrow osteogenic lineage covering a continuum of osteoblastic states (early osteoprogenitors, pre-osteoblasts, and osteoblasts) in diffusion map and gene expression analyses [37]. However, the molecular specification, morphology, and topographic distribution of the differentiation stages that link bmSSCs and bone-forming osteoblasts are still far from clear. Further work is required to address this point and to understand whether and how different biosynthetic and/or regulatory functions are potentially enacted by specific marrow osteogenic cell subsets.

### 4.2. Other Sources of Bone-Forming Cells

The application of sophisticated experimental approaches such as genetic manipulation, cell surface marker analysis, or a combination thereof, along with in vivo transplantation assays, has led to the identification of different populations of fetal and adult skeletal stem cells distinct from either early embryo progenitors and post-natal bmSSCs. In addition, the potential conversion of chondrocytes into bone-forming cells suggested many years ago [38,39], has been recently confirmed by solid in vivo experimental work [18]. As a consequence, a new paradigm is emerging in which multiple subsets of stem/progenitor cells with dedicated functions work together with differentiated cells at specific anatomic sites to ensure normal bone development and maintain bone homeostasis.

Chan and colleagues reported on an SSC population with a Podoplanin^+^/CD146^−^/CD73^+^/CD164^+^ profile that resides in the human fetal growth plate and post-natal femur, which gives rise to a hierarchy of lineage-committed progenitors [40]. A similar population, with a different surface phenotype, was previously identified by the same authors in mice [41]. This type of SSC differentiates into chondrocytes, especially in fetal life, and osteoblasts and marrow stroma, but it does not contribute to marrow adipocyte formation in post-natal life [40,41]. Debnath and colleagues [42] demonstrated the presence of skeletal stem cells in mouse and human periosteum (pSSCs). In mice, they are targeted by *Cathepsin K-Cre*, have a CD200^+^/CD105^−^ profile, and function in multiple skeletal sites such as long bones and calvaria. Their baseline activity is to form membranous bone, but they acquire an endochondral bone formation capacity in particular environments, such as in fracture callus [43]. Thus, pSSCs are currently thought to provide osteoblasts for modeling of the bone cortex and for the regeneration process that takes place during fracture healing [42,43]. Skeletal progenitors with proven in vivo osteogenic activity have been isolated from human cord blood based on the expression of CD146 and the presence of clonogenic activity [29]. These blood-borne osteoprogenitor cells are unlike other previously reported umbilical cord derivatives as shown, for example, by the expression pattern of *HOX* genes [44], and they share biological features with bmSSCs. However, their transcriptional profile and differentiation potency diverge markedly from those of post-natal bmSSCs. In contrast with bmSSCs that generate bone, stroma, and adipose tissues, CD146^+^ cord blood progenitor cells are inherently committed to cartilage and bone formation, thus mirroring the structure of the fetal skeleton [29]. Finally, evidence from different experimental systems demonstrates that in some circumstances, chondrocytes differentiate into bone-forming cells. This possibility initially emerged from histological studies in chickens and rats, showing that some hypertrophic chondrocytes resume proliferation, convert into a osteoblast-like phenotype, and participate in the deposition of the first bone matrix in developing long bones [39,45]. In parallel, explant culture experiments with chick embryos revealed the association of a lineage switch from hypertrophic chondrocytes to osteogenic cells with the process of asymmetric cell division [38]. A switch from chondrocytes to osteoblasts was also reported in membrane type 1-matrix metalloproteinase (MT1-MMP)-deficient mice within the transient cartilage anlages that form during the embryonic development of the skull [46]. More recently, lineage tracing studies in mice have shown the presence of osteoblast-like cells derived from hypertrophic chondrocytes in fetal and postnatal endochondral bones. These cells persist into adulthood and contribute to mature bone formation, participate in bone repair [18,47], and convert into bone marrow stromal cells and adipocytes [47,48]. In this context, it is interesting to note that chondroid rudiments generated by human BMSCs in a chondro-inductive culture medium form a bony collar in vitro upon switching to an appropriate osteo-inductive medium [49] and form a bone/marrow organoid in vivo upon heterotopic transplantation [50]. In the latter case, the establishment of a functional marrow cavity results from the reversion of fully differentiated chondrocytes into CD146^+^ BMSCs [50]. Altogether, these data suggest that the skeletal system is characterized by an intrinsic plasticity that allows for bidirectional transitions in specific circumstances not only between individual differentiated states, but also between progeny and progenitor. This behavior reflects a remarkable adaptive capacity and is likely rooted in specific molecular mechanisms and varying epigenetic landscapes.

**Table 1:** Human and mouse osteoprogenitor cell populations.

Human			
Source	Progenitor	Progeny (In Vivo)	Reference
Adult bone marrow	CD146^+^	Osteoblasts, adipocytes and stromal cells	[21]
Adult bone marrow	CD271^+^	Osteoblasts and stromal cells	[22]
Fetal bone marrow	Nestin^+^/CD45^−^/ Ter119^−^/CD31^−^/ PDGFRα^+^/CD51^+^	Osteoblasts, chondrocytes and adipocytes	[23]
Fetal and adult bone	PDPN^+^/CD146^−^/ CD73^+^/CD164^+^	Osteoblasts, chondrocytes and stromal cells	[40]
Periosteum	Lin^−^/CD90^−^/ CD200^+^/CD105^−^	Osteoblasts	[42]
**Mouse**			
**Source**	**Progenitor**	**Progeny (In Vivo)**	**Reference**
Limb bud mesenchyme	*Sox9-Cre*	Osteoblasts and chondrocytes	[51]
Adult bone marrow	a-SMA+	Osteoblasts	[52]
Adult bone marrow	PDGFRa^+^/Sca-1^+^/ CD45^−^/TER119^−^	Osteoblasts, adipocytes and stromal cells	[53]
Adult bone marrow	*Nestin-GFP*	Osteoblasts and chondrocytes	[54]
Adult bone marrow	CXCL12+	Osteoblasts and adipocytes	[55]
Adult bone marrow	*Osx-Cre*	Osteoblasts, adipocytes and stromal cells	[56]
Adult bone marrow	*LepR-Cre*	Osteoblasts, chondrocytes and adipocytes	[57]
Fetal and adult bone marrow	*Osx-Cre*	Osteoblasts and stromal cells	[58]
Adult bone marrow (metaphysis)	*Gremlin1-Cre*	Osteoblasts, chondrocytes and stromal cells	[59]
Growth plate	CD45^−^/Ter119^−^/Tie2^−^/ Thy1^−^/6C3^−^/CD51^+^/ CD105^−^/CD200^+^	Osteoblasts, chondrocytes and stromal cells	[41]
Adult bone marrow (methaphysis)	*Gli1-CreERT2*	Osteoblasts, chondrocytes and adipocytes	[60]
Adult bone marrow	CD45^−^/CD31^−^/ Sca1^+^/CD24^+^	Osteoblasts, chondrocytes and adipocytes	[61]
Periosteum	*Catepsin K-Cre*	Osteoblasts	[42]
Postnatal growth plate resting chondrocytes	*Pthrp-mCherry*	Osteoblasts, chondrocytes and stromal cells	[62]
Perichondrium, periosteum and bone marrow	*Hoxa11-CreERT2*	Osteoblasts, chondrocytes and adipocytes	[63]
Fetal bone marrow	*Kit-MerCreMer*	Osteoblasts, chondrocytes and stromal cells	[64]

## 5. Specification of Bone-Forming Cells

The process of osteoblastogenesis consists of a sequence of molecular events that leads to the activation of the secretory apparatus required to build up a mineralizing extracellular matrix. The generation of transgenic mice and parallel analysis of human skeletal disorders have provided major insights into the complex molecular and cellular network underlying osteoblast formation. Examples of critical players operating in this network are reported in the following paragraphs.

### 5.1. Transcription Factors

Lineage commitment within the skeleton is primarily dependent on three transcription factors; the sex-determining region Y-box (SOX9) [65], the runt-related transcription factor 2 (RUNX2)/core binding factor a1 (CBFA1) [66,67], and the peroxisome proliferator-activated receptor γ2 (PPARγ2) [68], which act as master regulators of chondrogenesis, osteogenesis and adipogenesis, respectively. During fetal development, skeletal progenitors have chondrogenic and osteogenic activity and their fate choice is dictated by the relative expression of SOX9 and RUNX2. After birth, osteogenesis and adipogenesis are the canonical differentiation pathways that emerge from bmSSCs, and the phenotypic specification is dependent on the relative expression of RUNX2 and PPARγ2.

RUNX2/CBFA1 is a member of the transcription factor family that shares the DNA-binding domains of homology with Drosophila Runt, and it is indispensable for bone formation. Its genetic ablation in mice causes a complete lack of bone [38,46], whereas haploinsufficiency in mice and humans leads to Cleidocranial Dysplasia (OMIM 119600), characterized by insufficient ossification of calvarial bones and clavicles [69]. In early developmental stages, RUNX2 stimulates the proliferation and osteogenic commitment of progenitors in membranous bones and in endochondral bones. In post-natal life, RUNX2 is required to induce an osteogenic program in bmSSCs at the expense of adipogenesis [70]. However, contrasting effects have been observed in transgenic mice with lack of function or over-expression restricted to different osteoblast developmental stages [71,72,73,74]. RUNX2 acts as an upstream inducer of other transcription factors, in particular, Osterix (OSX/SP7) [75], and it modulates the activity of multiple molecular pathways such as those induced by hedgehog (HH), fibroblast growth factors (FGFs), wingless type MMTV integration site (WNT), and parathyroid hormone-related peptide (PTHrP) [76], to which RUNX2 is linked via reciprocal regulation. In addition, RUNX2 has been reported to directly activate genes encoding bone matrix protein such as COL1A1, OPN, BSP, and OCN [71].

OSX is the second most important master gene of osteogenesis [75]. While RUNX2 is necessary to induce skeletal progenitor cell expansion by stimulating FGFR2 and FGFR3 [77] and segregation into the osteogenic lineage, OSX is required to complete the osteoblast differentiation process, as shown by the inability of *Osx*-null skeletal progenitors to deposit bone matrix [75]. In the adult skeleton, OSX continues to play a role in bone homeostasis by regulating the expression of osteoblastic and osteocytic genes [78]. Indeed, mutations in the *OSX* gene have been found in patients with an Osteogenesis Imperfecta phenotype (Osteogenesis Imperfecta type XII, OMIM 613849) [79]. However, as previously reported for RUNX2 [72], abnormal osteoblast maturation is also observed in mice overexpressing OSX [80], thus indicating that execution of a normal osteoblast differentiation program requires not only the presence, but also a proper level of expression of these two regulatory genes.

Other examples of transcription factors regulating osteoblastogenesis during development are Msh homeobox 2 (MSX2) and the basic helic-loop-helix containing factor, TWIST. MSX2 is particularly involved in the development of cranial bones where, in transgenic mouse models, it controls osteoprogenitor cell proliferation [81]. Accordingly, human patients with mutations causing enhanced DNA binding activity of MSX2 develop autosomal dominant craniosynostosis (OMIM 604757) [82], whereas *MSX2* haploinsufficiency is associated with enlarged parietal foramina (OMIM 168550) [83]. In contrast, TWIST plays a negative regulatory role in early skeletogenesis through the transient inhibition of RUNX2 [84], and its deficiency in humans results in increased bone formation in cranial sutures in Saethre-Chotzen syndrome with eyelid anomalies (OMIM 101400) [85]. Other examples of transcription factors modulating osteoblast formation are members of the activator protein 1 (AP1) family [86], MAF bZIP transcription factor (MAF) [87] and Forkhead box P1 (FOXP1) [88], which are involved primarily in the fate choices of post-natal bmSSCs into either osteoblast or adipocyte.

### 5.2. Molecular Pathways

Various secreted and cell-surface factors that activate complex molecular pathways participate in the regulation of osteoblast differentiation. WNT, HH, transforming growth factor βs (TGFβs) and bone morphogenetic proteins (BMPs) are well-known players, but others such as NOTCH, FGFs, and insulin-like growth factors (IGFs), are also involved [3,89]. Some of these pathways have a critical function throughout life, whereas others are more deeply involved in specific developmental phases or in post-natal homeostasis. In all cases, there is extensive cross-talk between the different pathways that operate in a coordinated manner to achieve proper osteoblast differentiation.

The WNT pathway includes multiple receptors, co-receptors, activators, inhibitors, and a central molecule, β-Catenin, based on which canonical (β-Catenin-dependent) and non-canonical (β-Catenin-independent) branches have been identified. Of these, the canonical cascade promotes osteogenic differentiation in pre- and post-natal life. Ablation studies in mice show that WNT-β-Catenin function in uncommitted, *Prrx1*-expressing skeletal progenitor cells does not affect *Runx2,* but it is necessary for the expression of *Osx* [90] and thereafter for further maturation into osteoblasts [91]. Of note, the lack of WNT-β-Catenin in early RUNX2^+^/OSX^+^ progenitor cells causes a fate shift, resulting in chondrocyte formation at ectopic sites (i.e., in place of osteoblasts) during development [90,91,92] and to increased marrow adiposity after birth [93]. Thus, WNT-β-Catenin activity is critical not only to initiate but also to maintain the osteoblastic program in early committed cells. At later stages of osteoblastic differentiation, WNT-β-Catenin signaling regulates the acquisition and maintenance of bone mass in multiple ways. It stimulates immature osteoblasts to fully differentiate and to complete normal bone matrix mineralization [94]; it establishes a positive osteogenic regulatory loop in which WNT secreted by mature cells induces further osteoblast formation by undifferentiated progenitors [95], and it modulates osteoblast–osteoclast interaction by regulating the expression of receptor activator of nuclear factor κ B ligand (RANKL) and osteoprotegerin (OPG) in osteoblasts [94,96]. Different genetic skeletal diseases are associated with mutations of genes encoding the *WNT* pathway members. For example, the Osteoporosis-Pseudoglioma Syndrome (OMIM 259770) is caused by loss-of-function mutation of the low-density lipoprotein receptor-related protein 5 (*LRP5*), which is a co-receptor involved in WNT signal transduction [97], while gain-of-function mutations of the same gene result in hyperostosis (OMIM 144750) [98]. Sclerosteosis (OMIM 269500) and van Buchem disease (OMIM 239100) associate with loss-of-function mutation in the gene encoding the inhibitory factor, Sclerostin [99], which inhibits the canonical WNT signaling pathway by binding to LRP5/LRP6 [100].

HH proteins and related molecules, such as the intracellular signaling protein, Smoothened (SMO) and the HH-responsive factors, Glioma-associated Oncogene Homolog 1, 2 and 3 (GLI1, GLI2 and GLI3), are essential for osteoblast formation during endochondral ossification. Studies in mice revealed that perichondral osteoprogenitor cells activate the osteoblastic program under the control of Indian Hedgehog (IHH) produced by neighboring pre-hypertrophic and hypertrophic chondrocytes [13]. *Runx2* has been recognized as the main mediator of this effect [17], although forced expression of *Runx2* alone does not restore endochondral bone formation in *Ihh*-null mice [101]. Similar to global *Ihh* ablation, the deletion of *Smo* (which is required for HH signal transduction in the perichondrium) targeted to chondrocytes by *Col2a1-Cre* prevents the formation of a normal bony collar and development of the primary spongiosa, and results in the appearance of chondrocytes in place of osteoblasts [102]. However, osteoblast formation is not affected when *Smo* is removed after osteogenic commitment by *Osx-Cre* [91]. Altogether, these data demonstrate that during skeletal development, IHH regulates osteoblastogenesis in endochondral ossification by targeting RUNX2 and other critical effectors, and that its function is required specifically in the very early stages of osteogenic commitment (before *Osx* expression). After birth, IHH continues to regulate osteoblast formation and activity, although its source(s) in the adult skeleton still remain(s) unclear. For example, *Gli1* haploinsufficient mice demonstrate that IHH participates in bone homeostasis by stimulating osteoblast maturation, while delaying osteocyte formation, and by modulating the expression of *Rankl* and *Opg* in osteogenic cells [103]. Consistent with its role in osteoblastogenesis, altered HH signaling is associated with multiple human skeletal diseases, such as Brachydactyly type 1A (OMIM 112500) [104] and Basal Cell Nevus Syndrome (OMIM 109400) [105]. In addition, abnormal activity of the HH pathway is involved in diseases with heterotopic ossification (as further discussed below).

The transforming growth factor β (TGFβ) family is comprised of multiple members including BMPs, TGFβs, Activin, and other related proteins. These factors act through a surface molecular complex made by type I and type II receptors that induce intracellular signals via either small mother against decapentaplegic (SMAD) proteins or through mitogen-activated protein kinase (MAPK). Many in vivo transgenic models have helped to dissect the function of the TGFβ super family members in osteoprogenitors during skeletal development and in homeostasis. TGFβ1, 2, and 3 are expressed during membranous and endochondral bone formation, but, with the exception of TGFβ2 [106], their ablation during development does not cause major abnormalities in skeletal development [107,108]. In contrast, in post-natal life, TGFβ1 released from the bone matrix at sites of osteoclast resorption has a critical regulatory role not only in osteoblast differentiation but also in the migration of bone marrow stromal osteoprogenitor cells through SMAD signaling and in coupling bone resorption and bone formation [109]. Accordingly, the abnormal migration and proliferation of bone-forming cells have been proposed as the pathogenetic mechanisms underlying the thickening of the skull and long bone diaphysis caused by mutations of the *TGFB1* gene in patients with Camurati-Engelman disease (OMIM 131300) [110]. BMP2 and BMP4 act during limb development on early osteoprogenitors by inducing *Osx* expression and the acquisition of an osteogenic phenotype and are therefore required to form cortical and trabecular bone [111]. At later developmental stages, the TGFβ/BMP pathways control multiple cell functions. For example, in Osx^+^ differentiating osteoblasts, SMAD4 interacts with RUNX2 and WNT-β-Catenin to control the apoptosis process and the expression of collagen-processing enzymes that ensure the structural integrity of the extracellular bone matrix [112]. In mature, *Ocn*-expressing osteoblasts, SMAD-mediated signaling controls cell proliferation, bone matrix deposition, and the expression of *Rankl*/*Opg* [113]. Interesting results on the molecular targets and mechanisms of action of the TGFβ/BMP system in osteoblastogenesis come also from in vitro studies with human and mouse cells. These studies provide a detailed analysis of the gene expression profile of immortalized human bone marrow stromal cells during BMP-dependent osteogenic differentiation [114], and they show that BMP2 is able to stimulate *Osx* expression through a RUNX2-dependent and RUNX2-independent, MSX2-mediated, mechanism [115]. Furthermore, they reveal that BMPs act via multiple avenues involving gene expression, post-transcriptional (miRNA-mediated) modulation [116], and post-translational modifications [117]. Finally, it must be noted that some BMP members are very powerful osteoinductive factors and are able to induce an osteogenic phenotype in non-skeletal cells that would never make bone in physiological conditions [118]. This effect underlies the phenotype of Fibrodysplasia Ossificans Progressive (OMIM 135100), which is a skeletal disorder caused by mutations in the BMP type I receptor, *ACVR1*, and characterized by skeletal malformations and ectopic bone formation [119].

### 5.3. Gsα-cAMP Signaling Pathway

Many local and systemic factors such as cytokines, growth factors, neurotransmitters, and hormones modulate osteoblastogenesis by acting on common intracellular signaling transducers that target specific effectors, while interacting with the molecular pathways reported above. The Gsα/cAMP system transduces signals from peptide hormones (mainly PTH), prostaglandins and neurotransmitters, which bind G protein coupled receptors (GPCRs) on the cell surface. The α subunit of the stimulatory G protein (Gsα) is encoded at the *GNAS* locus, and following receptor stimulation, it increases the intracellular level of cAMP [120]. The importance of Gsα in the regulation of skeletal development and homeostasis is highlighted by the wide range and complex phenotype of skeletal disorders caused by mutations of *GNAS* that alter its function. Reduced Gsα activity, due to heterozygous loss-of-function mutations, leads to abnormal osteogenesis in the embryo and results in Albright’s Hereditary Osteodystrophy (OMIM 103580), which is a combination of short stature, brachydactyly, brachymetacarpia and subcutaneous ossification [121,122] or in Progressive Osseous Heteroplasia (OMIM 166350) [123]. In contrast, increased Gsα activity, caused by gain-of-function mutations that result from abnormal post-zygotic methylation of *GNAS* [124], severely affects the homeostasis of the post-natal skeleton, leading to FD [125]. Dissecting the activity of Gsα/cAMP in bone-forming cell commitment, differentiation, and function is a complex issue due to multiple stimulators and extensive cross-talk with other molecular pathways. However, significant insights emerge from transgenic mice with Gsα dysfunction restricted to different skeletal developmental phases or osteoblast maturational stages. In embryonic life, Gsα controls the process of osteogenesis and its spatial distribution by interacting with WNT/β-Catenin and HH. Regard et al. showed that *Gnas* ablation in mouse embryonic cells expressing the *Prrx1*, *Twist2*, or *Tfap2a* promoter causes skeletal abnormalities by reducing the expression of WNT target genes and β-Catenin in osteoprogenitor cells, and leads to heterotopic ossification by altering the expression of *Gli2* and *Gli3* in soft tissue cells. Thus, in embryonic life, Gsα activity ensures a normal spatial pattern of ossification by stimulating the WNT pathway at the prospective skeletal sites and by inhibiting the HH system outside the osteogenic territory [126]. Accordingly, the interaction between Gsα and HH is critical for the process of membranous ossification that underlies the development of cranial bones, wherein Gsα modulates the HH pathway in a ligand independent manner [127]. In committed osteoprogenitor cells, Gsα regulates WNT to direct the fate and to control the different phases of osteoblast differentiation. Ablation of Gsα in mouse *Osx* promoter-expressing cells leads to a high level of expression of the WNT inhibitors, Sclerostin and dickkopf1 (DKK1) [128]. This results in a phenotype characterized by expanded bone marrow adipocytes [129], reduced osteoblasts, and a decreased amount of trabecular and cortical bone [128]. Interestingly, the effect on the osteoblast compartment is due not only to the impaired commitment of progenitor cells but also to accelerated differentiation of osteoblasts into osteocytes, which in turn, causes structural abnormalities (deposition of woven bone with a disorganized pattern of mineralization) in skeletal segments of different embryological origins. Thus, Gsα enhances osteoblast differentiation in early phases but reduces it at later stages, and through these opposite effects, it controls both the amount and the quality of the newly formed bone [128]. This is further supported by studies showing that Gsα expression decreases during osteogenic differentiation in parallel with increased expression, phosphorylation, and DNA binding of RUNX2 [130]. Gsα activity in fully differentiated osteogenic cells affects bone mass both directly by controlling osteoblast functions, and indirectly by controlling osteoclast recruitment and bone turnover. A lack of Gsα in mouse cells expressing the 2.3kb *Col1a1* promoter, which specifically defines the functional state of osteoblasts, causes a transgenic phenotype characterized by defective formation of primary spongiosa, reduced bone length, and low trabecular bone volume associated with thickening of cortical bone due to reduced bone resorption [131]. Ablation of Gsα in murine osteocytes expressing the *Dmp1* promoter causes an osteopenic phenotype that appears earlier in cortical than trabecular bone, and it is explained, at least in part, by the increased expression of the WNT inhibitor, Sclerostin [132]. In keeping with these results, overactivity of Gsα in osteoblasts due to gain-of-function mutations [133] or overexpression of the wild-type gene [134] results in mice with a high bone mass phenotype. Interestingly, in the gain-of-function mutation model, bone-forming cells display a markedly different level of activity across the skeleton, which results in severe bone deformities. This non-uniform response to the transgene suggests the existence of a variable response of osteoblasts to endogenous Gsα. However, it remains to be assessed whether this is due to the intrinsic difference among osteoblasts at different anatomical sites or to the presence of extrinsic, site-specific modulators. In addition, mice with activating Gsα mutation show abundant deposition of periosteal bone. This phenotype is not mirrored in mice with constitutive activation of the PTH/PTHrP receptor (PPR) under the same osteoblast promoter and in the same genetic background [135], thus suggesting that the action of Gsα on the periosteum is not dependent on PTH stimulation.

**Figure 3:**
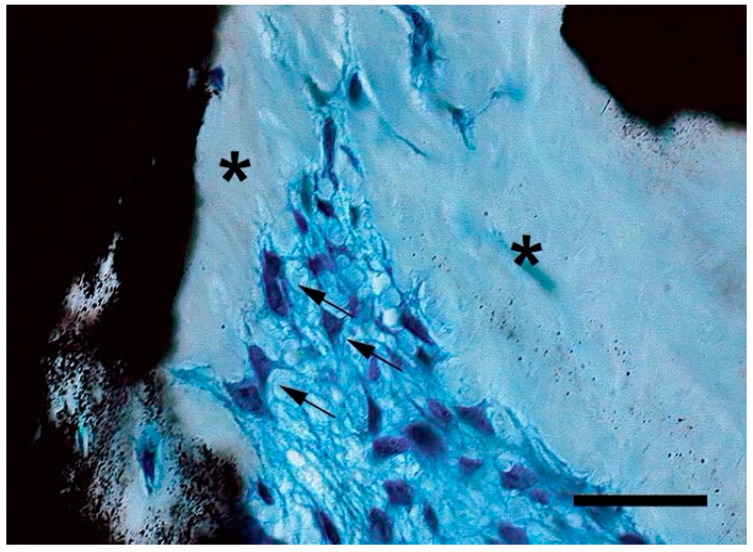
Abnormally shaped (i.e., retracted, arrows) osteoblasts and excessive unmineralized bone matrix (asterisks) in Fibrous Dysplasia (FD) bone. Formalin-fixed undecalcified plastic-embedded sample, Von Kossa-Methylene Blue. Bar: 50 µm.

Finally, interesting data on the function of Gsα in osteogenic cells are provided by mouse models in which activating Gsα mutations are randomly integrated into the genome [136]. Activating mutations of Gsα are never inherited in humans and are lethal in mice when expressed at the *Gnas* locus [137]. In contrast, mice with the mutated Gsα cDNA sequence expressed outside of the *Gnas* locus undergo normal skeletal development and show the same life span as their wild-type littermates. However, osteogenic differentiation is severely compromised in post-natal bmSSCs, leading to a skeletal phenotype that faithfully reproduces human FD [136]. In addition to demonstrating that over-activity of Gsα may be compatible with normal skeletal morphogenesis and fetal bone growth in certain circumstances; e.g., in the presence of a normal function of other *GNAS* products, these mice confirm that FD is a disease that selectively affects post-natal bmSSCs and therefore represents a useful model to study the biology and function of this specific stem cell cohort. Indeed, FD demonstrates that the adipogenic capability and hematopoiesis-supporting activity of bmSSCs, as well as their osteogenic differentiation, are strictly regulated by Gsα. In FD lesions, BMSCs/SSCs show increased expression of *c-fos* [138], enhanced proliferation [139], accelerated commitment to pre-osteoblasts, and differentiation into abnormally shaped osteoblasts (Figure 3). The latter produce an abnormal bone matrix with distinct structural features (collagen bundles running perpendicular to the forming surface, i.e., Sharpey’s fibres), chemical composition (enrichment of ON and low levels of OPN and BSP), and reduced mineralization [33,140,141,142].

### 5.4. Epigenetic Regulation and miRNAs

Osteoblastogenesis, as with any other cell differentiation pathway, is based on a continuous flow of information between genes (constant) and gene expression patterns (variable). This flow is modulated by mechanisms that act at transcriptional and/or translational levels and can result in inheritable effects without changing the sequence of the DNA.

*Epigenetic regulation* is based on chemical modifications of histone and DNA and modulates gene expression by affecting the functional state of the chromatin. Epigenetic mechanisms start to operate in early stages of skeletal formation. For example, methylation of the Histone 3 Lysine 27 (H3K27), which is an inhibitory epigenetic mark, is one of the mechanisms through which the expression of BMP/WNT members is modulated during osteogenic differentiation of early mouse mesenchymal progenitor cells [143]. However, the most intriguing aspects in bone epigenetics at this time are deciphering the physiological epigenetic signature of post-natal BMSCs/SSCs [144] and changes during skeletal aging. Overall, available data point to a major role of histone modification and DNA methylation in reduced osteoblastogenesis and increased adipogenesis that characterize human and mouse BMSCs/SSCs from aged donors [88,145]. Of note, a clear assessment of the epigenetic profile of post-natal BMSCs/SSCs is highly desired not only for a deeper understanding of bone biology and bone diseases such as osteoporosis, but also to refine and improve the protocols for ex vivo expansion of these cells before their use in bone tissue engineering. Indeed, epigenetic changes and other senescence-related mechanisms, e.g., oxidative stress [146], have been demonstrated to occur in BMSCs/SSCs both in vivo and in vitro [88,145], with consequent negative impact on their differentiation capacity and on their ability to regenerate bone.

*MicroRNAs* (miRNAs) are 20–25-nucleotide, single-stranded noncoding RNAs that regulate gene expression mainly, but not exclusively, at the post-transcriptional level, either by degrading mRNAs or by inhibiting their translation. miRNAs that have been shown to modulate osteoblast differentiation in vitro and/or bone formation in vivo are collectively named OsteomiRs and may be classified according to their inhibitory or stimulatory function, or based on their specific targets. Only a few examples of OsteomiRs are provided here, and a more exhaustive review may be found, for example, in Moghaddam and Neshati [147]. miR-133 and miR-135 are interesting examples of inhibitory miRNAs that are involved in the regulation of early osteogenic commitment by BMP2 [148]. Interestingly, miR-133 promotes myogenic differentiation, and this suggests that its modulation could represent one of the mechanisms by which BMP modulates the emergence of different, tissue-specific phenotypes in early connective tissue progenitor cells. miR-206 regulates the differentiation of osteoprogenitors during embryo development and has been associated with some post-natal skeletal diseases. In the mouse embryo, it is highly expressed in the perichondrium where it likely contributes to maintain perichondral osteoprogenitor cells in an undifferentiated state by targeting *Cx43* [149]. In addition, experimental studies in rats show that the miR-206/CX43 axis is involved in abnormal osteoblast function associated with steroid-induced femoral head necrosis [150]. miR-138 is an inhibitory miRNA that affects the differentiation of human BMSCs/SSCs by targeting the focal adhesion kinase pathway [151]. miR-196 and miR-188 are inhibitory OsteomiRs that are up-regulated in human and murine osteoprogenitors in an age-dependent fashion and may contribute to the low bone mass associated with senescence [152,153].

### 5.5. Lamins and Autophagy

Proper structural assembly of cell compartments, as well as timely degradation of cell dysfunctional components, may affect many biological processes including osteoblast differentiation. Lamins are type V intermediate filament proteins that line the nuclear membrane on its nucleoplasmic side, and perform mechanical functions while providing a substrate for binding of proteins and DNA for the regulation of gene expression and intracellular signaling pathways. The accumulation of immature (prelamin A) or mutant forms (progerin) of lamin A causes Hutchinson-Gilford Progeria Syndrome (OMIM 176670), Mandibuloacral Dysplasia with type A (OMIM 248370) or type B (OMIM 608612) Lypodystrophy, Restrictive Dermopathy (Lethal, OMIM 275210), and other disorders collectively named progeroid laminopathies, and they are characterized by severe bone pathology [154]. Indeed, lamins participate in the regulation of osteogenesis in different ways. Lamins A/C are involved in the balance between osteoblast and adipocyte differentiation in mouse and human systems. Liu et al. showed that in *Osx*-expressing mouse progenitors, vascular endothelial growth factor (VEGF) stimulates osteoblastic differentiation in a RUNX2-dependent manner by establishing an intracrine mechanism with lamins A/C [155], whereas Swift et al. showed that an increased expression of lamin A and consequent nuclear stabilization mediate the effect of tissue stiffness and stress on the lineage determination of human bone marrow stromal stem/progenitor cells [156]. In the latter, lamins A/C regulate osteoblast differentiation by also affecting the binding activity of RUNX2 [157] and the entry of β-Catenin into the nucleus [158]. The stimulatory function of lamin A is maintained in its immature and mutated forms. In human marrow osteoprogenitor cells grown in basal conditions, progerin expression increases the levels of OPN [159] whereas prelamin A induces an osteogenic secretome [160]. Although counterintuitive, these data suggest that an early, accelerated osteogenesis is part of the normal process of senescence in progeroid laminopathies that contributes to the unbalanced homeostasis of skeletal tissues and to the pathological process [160]. In agreement with in vitro studies, low bone mass, deformities, fracture and reduced numbers of osteogenic cells are observed in transgenic mice with disrupted lamin A gene or progerin expression [161,162].

*Autophagy* is an important mechanism for balancing sources of energy at critical times during development and in response to nutrient stress. Clinical observations have shown a link between autophagy inducers and bone health [163], and experimental evidence demonstrate that the perturbation of autophagy causes bone cell dysfunction. In addition to in vitro studies showing the role of autophagy in the survival of BMSCs/SSCs to stress [164], many transgenic models demonstrate the importance of this process in osteoblast differentiation and function. An age-dependent increase in bone mass and bone mineral density is observed in mice with global genetic truncation of NBR1, which is an autophagy receptor involved in targeting ubiquitinated proteins for degradation [165]. This skeletal phenotype results from an increased number of marrow progenitor cells and osteoblasts mediated by p38 MAPK signaling. Knock-out of autophagy-related factors targeted to bone cells directly confirms their function in bone homeostasis. Knock-out of *Fip200*, a component of the autophagy-related complex, ULKs-ATG13-FIP200, in osteogenic cells by either *Osx-Cre*, *Cola1-3.6-Cre* or *Cola1-2.3-Cre* causes an osteopenic phenotype associated with defective terminal differentiation of bone marrow progenitor cells into osteoblasts [166]. A similar low bone mass phenotype with abnormal osteoblast differentiation, matrix deposition, and transition from osteoblasts to osteocytes is obtained by the deletion of autophagy related 7 gene (*Atg7*), which is an E1-like enzyme essential for autophagy, by either *Osx-Cre* [167], *2.3 kb Col1a1-Cre* [168] or *Dmp1-Cre* [169]. In addition to affecting osteoblast differentiation, autophagy seems to be deeply involved in extracellular matrix mineralization. Interestingly, Nollet et al. reported that the presence of autophagic vesicles containing needle-like crystal structures in osteoblasts suggest that they could serve as vehicles to secrete pre-formed apatite crystals into the extracellular space [170].

### 5.6. Osteoclasts as Modulators of Osteoblastogenesis

Osteoblast differentiation and function are modulated by multiple cell types within skeletal and non-skeletal lineages, such as chondrocytes, osteocytes, endocrine cells, neurons, and immune cells [4,171]. The participation of osteoclasts in the regulation of osteoblastogenesis and osteogenesis was initially suggested by Martin and colleagues [172]. Since then, multiple studies have provided new insights into the role of osteoclasts in bone remodeling and in pathological conditions characterized by high bone turnover. During bone remodeling, the balance between bone formation and bone resorption, which is critical for the maintenance of bone mass, relies on mechanisms that couple osteoclast activity with recruitment, differentiation of osteoprogenitor cells, and with the function of differentiated osteoblasts. These mechanisms have long been thought to essentially coincide with the release of anabolic factors, such as TGFβ1, from the resorbed bone matrix. However, it is now clear that osteoclasts participate in a direct manner, i.e., independent of their resorption activity in the coupling process by secreting regulatory factors, by shedding membrane-coated vesicles containing proteins and miRNAs, and by establishing cell–cell contacts with osteogenic cells [173]. Secreted molecules such as WNT10B, BMP6, the GP130-signaling cytokine, cardiotrophin-1 (CT-1), collagen triple helix repeat containing 1 (CTHRC1), leukemia inhibitory factor (LIF) and RANK, which is delivered by vesicles and promotes RANKL reverse signaling [174], act as stimulators of osteogenesis. Inhibitory factors include miRNAs [175], whereas molecules such as sphingosine-1-phosphate (S1P) may have different effects according to the differentiation stages of osteoprogenitor cells [176]. Examples of membrane-bound proteins that participate in cell–cell contacts with osteoblasts are Ephrin B2 (interacting with EPH receptor B4) [177] and semaphorin D (binding Plexin B1), through which osteoclasts stimulate and inhibit bone formation, respectively [178]. Very recent data show that at the end of bone resorption, osteoclasts divide into smaller daughter cells termed “osteomorphs”. Interestingly, transcriptome analysis suggests that “osteomorphs” also participate in the regulation of bone structure and function through the upregulation of genes that are not expressed by their parental osteoclasts [179]. Regardless of the precise mechanisms, the role of osteoclasts and, likely, “osteomorphs” in modulating the differentiation and function of bone-forming cells has important implications. The most intuitive is that recognition may lead to the identification of novel potential therapeutic targets for diseases of bone remodeling such as osteoporosis. However, it should not be overlooked that it also provides a new perspective for the comprehension and even the treatment of bone diseases with high osteoclast numbers such as FD or giant cell tumors of bone. For example, by using a mouse model of FD, we have shown that osteoclasts contribute to the pathogenesis of the disease by preventing the differentiation of osteogenic cells within FD lesions, independent of their resorption activity. This may explain why the inhibition of RANKL in transgenic FD mice rapidly converts the osteogenic fibrous-like tissue into hyper-mineralized bone [180], while bisphosphonates, which inhibit bone resorption but not osteoclast formation, do not modify the histopathology of the disease in both transgenic mice [181] and FD patients [141,182].

## 6. Conclusions

**Figure 4:**
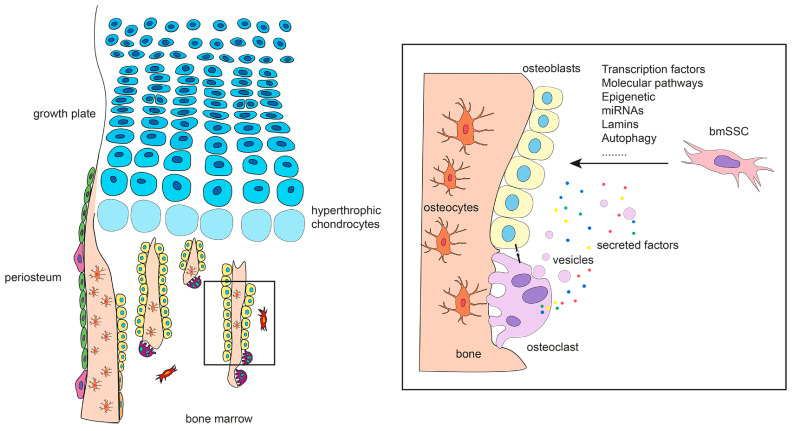
Left panel: Long bone skeletal compartments that host different subsets of stem/progenitor cells. Hypertrophic chondrocytes may also differentiate into bone forming cells. Right panel: Osteoclasts modulate osteogenic differentiation and osteoblast function by secreting regulatory factors and membrane-coated vesicles and by establishing cell–cell contacts with osteogenic cells.

The last years have witnessed a remarkable expansion of knowledge on the cell origin of osteoblasts and on the regulatory signals provided by molecules, signaling pathways, and cell processes during the specification of the osteoblastic lineage. Even though the role of all the cellular and molecular players identified is fairly well known, some important questions remain to be addressed. For example, it must be clarified whether, and to what extent, the different types of osteoprogenitors in the different skeletal compartments (Figure 4, left panel) collaborate before and after birth, and what signals orchestrate their function. Similarly, further investigation is required to define conclusively the phenotypic and functional steps through which undifferentiated cells are shaped into functionally active osteoblasts and how each step is regulated by systemic and local factors. In this context, the emergent role of previously unrecognized players such as osteoclasts (Figure 4, right panel) provides a novel outlook on bone homeostasis and on a variety of bone diseases. Finally, the osteoblast-specific molecular signature also requires further analyses to assess potential differences among osteoblast populations from different sites and cellular origins.

## Data Availability

No new data were created in this study. Data sharing is not applicable to this article.
